# Rnd3-induced cell rounding requires interaction with Plexin-B2

**DOI:** 10.1242/jcs.192211

**Published:** 2016-11-01

**Authors:** Brad McColl, Ritu Garg, Philippe Riou, Kirsi Riento, Anne J. Ridley

**Affiliations:** Randall Division of Cell and Molecular Biophysics, King's College London, New Hunt's House, Guy's Campus, London SE1 1UL, UK

**Keywords:** Plexin, Rho GTPase, Actin cytoskeleton, Cell shape

## Abstract

Rnd proteins are atypical members of the Rho GTPase family that induce actin cytoskeletal reorganization and cell rounding. Rnd proteins have been reported to bind to the intracellular domain of several plexin receptors, but whether plexins contribute to the Rnd-induced rounding response is not known. Here we show that Rnd3 interacts preferentially with plexin-B2 of the three plexin-B proteins, whereas Rnd2 interacts with all three B-type plexins, and Rnd1 shows only very weak interaction with plexin-B proteins in immunoprecipitations. Plexin-B1 has been reported to act as a GAP for R-Ras and/or Rap1 proteins. We show that all three plexin-B proteins interact with R-Ras and Rap1, but Rnd proteins do not alter this interaction or R-Ras or Rap1 activity. We demonstrate that plexin-B2 promotes Rnd3-induced cell rounding and loss of stress fibres, and enhances the inhibition of HeLa cell invasion by Rnd3. We identify the amino acids in Rnd3 that are required for plexin-B2 interaction, and show that mutation of these amino acids prevents Rnd3-induced morphological changes. These results indicate that plexin-B2 is a downstream target for Rnd3, which contributes to its cellular function.

## Abstract (precis)

**Summary:** The Rho GTPase Rnd3 is known to alter cell shape and the actin cytoskeleton. Here, the semaphorin receptor plexin-B2 is identified as a new Rnd3 partner that mediates its effects on cell shape.

## INTRODUCTION

The three Rnd proteins, Rnd1, Rnd2 and Rnd3, are members of the Rho family of small GTPases. They are atypical because they lack intrinsic GTPase activity and exist only in the GTP-bound form (Riou et al., 2010). They are instead regulated by reversible phosphorylation at their C-termini, which promotes binding of 14-3-3 proteins. The 14-3-3 proteins in turn extract Rnd proteins from membranes and inactivate them by translocating them to the cytosol (Riou et al., 2013).

In many mammalian cell types, Rnd1 and Rnd3 act antagonistically to RhoA by inducing loss of actin stress fibres and cell rounding (Guasch et al., 1998; Nobes et al., 1998). By contrast, Rnd2 does not induce cell rounding, probably because its localization is different to that of Rnd1 and Rnd3 (Oinuma et al., 2012). Rnd2 and Rnd3 play distinct roles in cortical neuronal migration during mouse development, in part because their expression is regulated by different transcription factors (Pacary et al., 2011). Interestingly, in endothelial cells, both Rnd2 and Rnd3 induce stress fibre assembly by upregulating RhoB expression (Gottesbuhren et al., 2013).

Several proteins that interact with Rnd proteins and could mediate their effects on cell shape and motility have been reported (Riou et al., 2010). The first to be identified was p190RhoGAP, which downregulates RhoA activity. Rnd3 interaction with p190RhoGAP stimulates its activity and induces loss of stress fibres (Wennerberg et al., 2003). Rnd3, but not Rnd1 or Rnd2, also binds to and is phosphorylated by the serine/threonine kinases ROCK1 and PKC (Madigan et al., 2009; Riento et al., 2005). Rnd1 interacts with the transmembrane receptor plexin-B1, and is reported to promote plexin-B1-induced RhoA activation (Oinuma et al., 2003; Rohm et al., 2000). Plexins are well known as neuronal guidance receptors that are activated by semaphorin ligands and frequently promote growth cone collapse (Püschel, 2007). They also contribute to multiple aspects of mammalian development, as well as angiogenesis, cancer progression and immune responses (Perälä et al., 2012; Worzfeld et al., 2014).

**Fig. 1.**
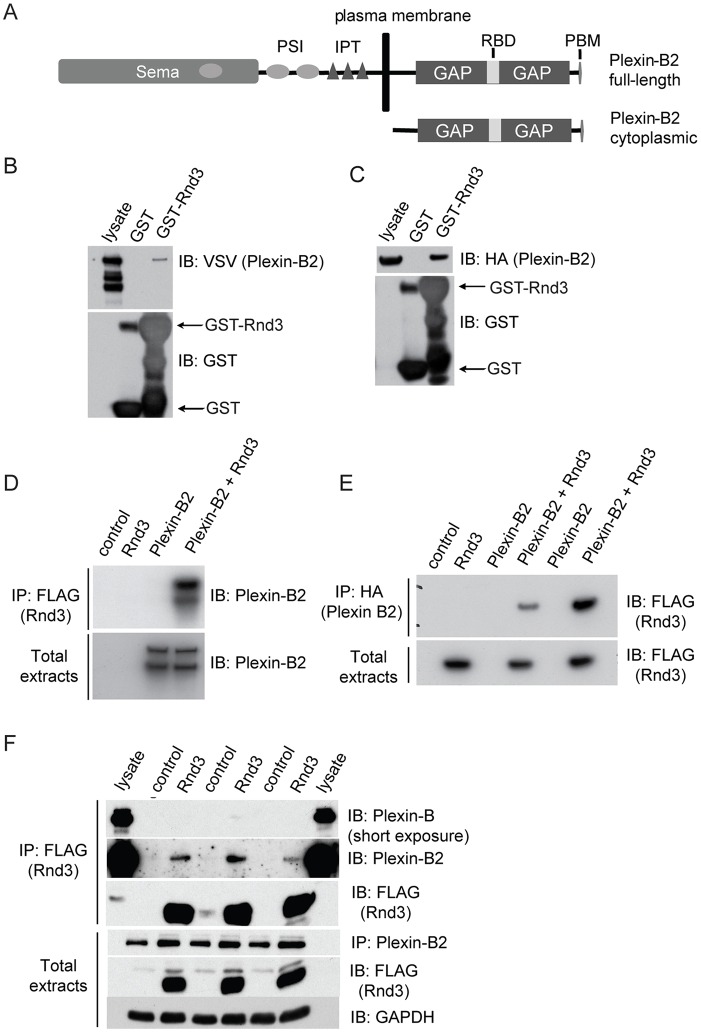
**Rnd3 interacts with plexin-B2.** (A) Schematic representation of plexin-B2 (full-length and cytoplasmic region) showing the extracellular Sema domain, plexins–semaphorins–integrins (PSI) domains and immunoglobulins–plexins–transcription factor (IPT) domains, and the intracellular split GAP domain, Rho-binding domain (RBD) and C-terminal PDZ-binding motif (PBM). (B,C) COS7 cells were transfected with expression vectors encoding VSV-epitope-tagged full-length plexin-B2 (B) or HA-epitope–plexin-B2-cyto (C). Cell lysates were incubated with GST or GST–Rnd3 on glutathione–Sepharose beads. Bound proteins were analysed by immunoblotting with the indicated antibodies. (D,E) Lysates of COS7 cells expressing full-length plexin-B2 (D) or HA–plexin-B2-cyto (E) and FLAG–Rnd3 were immunoprecipitated with anti-FLAG or anti-HA antibody, followed by immunoblotting. Full-length plexin-B2 migrates as a doublet at around 180 kDa. (F) Lysates of HeLa cells expressing FLAG–Rnd3 or transfected with empty vector (control) were immunoprecipitated with anti-FLAG antibody, followed by immunoblotting for endogenous plexin-B2. Results from three independent experiments are shown in the blot. All other blots are representative of three independent experiments.

Plexins have a Rho-binding domain (RBD) in the middle of a split GAP domain, which is reported to interact with R-Ras and act as a GAP for Rap1 (Fig. 1A) (Fansa et al., 2013; Pascoe et al., 2015). Several Rho GTPases have been reported to bind to the RBD of plexin-B1 in addition to Rnd1, including Rac1, RhoD, Rnd2 and Rnd3 (Fansa et al., 2013). Rnd proteins can also bind to other plexins, such as plexin-A1 and plexin-D1 (Rohm et al., 2000; Uesugi et al., 2009). Here, we report that Rnd3 interacts preferentially with plexin-B2 compared with plexin-B1 and plexin-B3, and map the amino acids in Rnd3 required for plexin-B2 interaction. Rnd3 interaction with plexin-B2 is required for it to induce cell rounding, indicating that it is a target for Rnd3 activity.

## RESULTS

### Rnd3 interacts with plexin-B2

To identify novel Rnd3 targets, a yeast two-hybrid screen was carried out with Rnd3 lacking the C-terminal CAAX box, to prevent farnesylation (Foster et al., 1996). This screen identified p190RhoGAP-B (ARHGAP5), a known Rnd3 interacting partner (Wennerberg et al., 2003), the plexin-B2 intracellular domain, interferon transmembrane protein 3 (IFTM3) and the potassium channel transmembrane domain 10 (KCTD10, also known as BACURD3) (Table S1). Because plexins are known to interact with Rnd proteins (Azzarelli et al., 2014; Hota and Buck, 2012; Oinuma et al., 2003), we investigated the interactions of plexin-B proteins with Rnd proteins in more depth.

Plexin-B2 is a type 1 transmembrane receptor, with an extracellular region and intracellular (cytoplasmic) region (Fig. 1A). Full-length VSV-tagged plexin-B2 and the HA-tagged cytoplasmic region of plexin-B2 (plexin-B2-cyto) interacted with recombinant glutathione S-transferase (GST)-tagged Rnd3 in pulldown assays (Fig. 1B,C), indicating that the interaction of Rnd3 with the cytoplasmic region of plexin-B2 is direct. Plexin-B2-cyto and full-length plexin-B2 co-immunoprecipitated with exogenous Rnd3 from COS7 cells (Fig. 1D,E). Endogenous plexin-B2 co-immunoprecipitated with exogenous Rnd3 (Fig. 1F). Taken together, these results indicate that plexin-B2 associates with Rnd3. This is probably a direct interaction, similar to that seen in the crystal structure of plexin-B1 with Rnd1 (Tong et al., 2009).

### Interactions between different Rnd and plexin-B family members

**Fig. 2.**
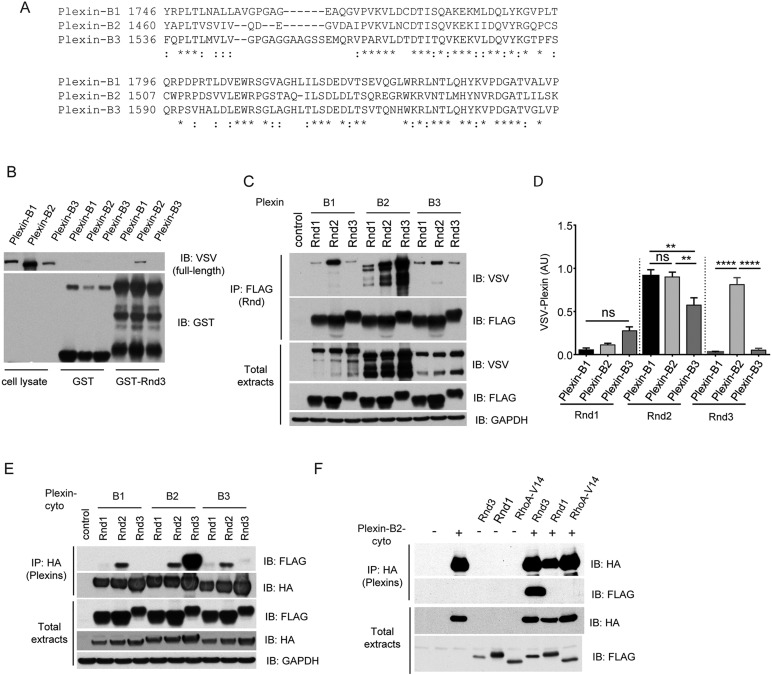
**Comparison of Rnd protein interactions with plexin-B1, plexin-B2 and plexin-B3.** (A) Alignment of RBD sequences from plexins B1, B2 and B3; * indicates identical amino acids, : indicates similar amino acids in all three sequences. (B) Lysates from COS7 cells expressing full-length VSV–plexin-B1, plexin-B2 or plexin-B3 were incubated with GST or GST–Rnd3 bound to glutathione–Sepharose beads. The bound proteins were analysed by immunoblotting with anti-VSV antibody. (C) Lysates of COS7 cells co-expressing VSV-tagged plexins B1, B2 or B3 and FLAG-tagged Rnd1, Rnd2 or Rnd3 were immunoprecipitated with anti-FLAG antibody, followed by immunoblotting with anti-VSV antibody. (D) Relative levels of immunoprecipitated VSV–plexins (mean±s.e.m., *n*=3; **P<0.01; ****P<0.0001); AU, arbitrary units. (E) Lysates of COS7 cells co-expressing HA–plexins B1, B2 or B3-cyto and FLAG-tagged Rnd1, Rnd2 or Rnd3 were immunoprecipitated with anti-HA antibody, followed by immunoblotting with anti-FLAG antibody. (F) Lysates from COS7 cells expressing HA–plexin-B2-cyto and FLAG–Rnd3, FLAG–Rnd1 or FLAG–RhoA-G14V were immunoprecipitated with anti-HA antibody, followed by immunoblotting. All blots are representative of three independent experiments.

The RBD domains of plexins B1, B2 and B3 are highly homologous (Fig. 2A) (Tong et al., 2009). We therefore tested whether Rnd3 was able to interact with either plexin-B1 or plexin-B3. GST–Rnd3 but not GST alone pulled down full-length plexin-B2 as expected, but very little plexin-B3 or plexin-B1 (Fig. 2B). Similarly, Rnd3 co-immunoprecipitated with full-length plexin-B2, but much less with plexin-B3 or plexin-B1 (Fig. 2C; quantified in Fig. 2D). These results show that Rnd3 interacts predominantly with plexin-B2. Plexin-B2 interacted with Rnd2 as well as Rnd3, but only weakly with Rnd1 and not detectably with constitutively active RhoA-G14V (Fig. 2C–F). Full-length and cytoplasmic regions of plexins B1, B2 and B3 all interacted similarly with Rnd2, whereas Rnd1 interacted only weakly with all three plexins (Fig. 2C–F).

**Fig. 3.**
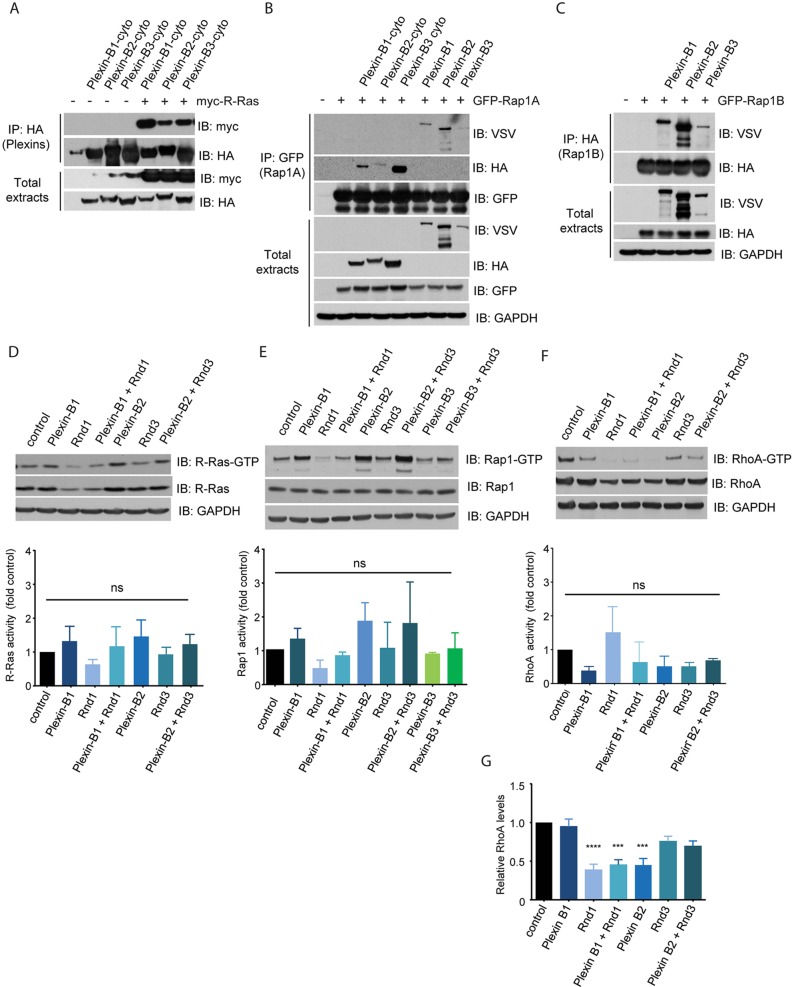
**Rap1A, Rap1B and R-Ras interact with plexin-B1 and plexin-B2.** (A–C) COS7 cells were transfected with expression vectors encoding VSV-tagged full-length plexins B1 or B2 or B3 or HA-tagged plexin-B1-cyto or plexin-B2-cyto and (A) myc-R-Ras, (B) GFP–Rap1A or (C) HA–Rap1B. Cell lysates were immunoprecipitated (IP) and western blotted with the indicated antibodies. All blots are representative of three independent experiments. (D–G) COS7 cells were transfected with expression vectors encoding HA-tagged plexins B1, B2 or B3 (cytoplasmic regions) and/or FLAG-tagged Rnd1, Rnd2 or Rnd3 and (D) myc-R-Ras, (B) GFP–Rap1A or (F,G) GFP–RhoA. After 6 h, the medium was replaced with medium containing 1% FCS and left for 18 h. After 24 h, cells were lysed and GTPase activity levels analysed. The graphs show (D) R-Ras, (E) Rap1A or (F) RhoA activity levels normalized to the respective total protein, relative to control, or (G) total RhoA levels normalized to GADPH, relative to control. Values are mean±s.e.m. of three independent experiments; ***P<0.001; ****P<0.0001; ns, not significant; one-way ANOVA with Tukey posthoc test for multiple comparisons.

Plexin-B1 has been reported to interact with and act as a GAP for the small GTPases R-Ras or Rap1 (Oinuma et al., 2004; Wang et al., 2012). We therefore tested the effects of Rnd proteins on plexin-B interactions with R-Ras and the two closely related Rap1 proteins, Rap1A and Rap1B. Similar to plexin-B1, we found that plexin-B2 and plexin-B3 associated with R-Ras, Rap1A and Rap1B (Fig. 3A–C). To determine whether Rnd proteins and/or plexin-B proteins affect R-Ras or Rap1 activity, active GTP-bound R-Ras and Rap1 (antibody recognizing Rap1A and Rap1B) were pulled down from cells expressing exogenous plexins and/or Rnd proteins. No significant changes in R-Ras activity or Rap1 activity were detected when Rnd1, Rnd3 and/or plexin-B1 or plexin-B2 were co-expressed (Fig. 3D,E). These results indicate that Rnd1 and Rnd3 proteins do not stimulate plexin-B1 or plexin-B2 GAP activity. This is consistent with the lack of change in the structure of the plexin-B1 GAP domain upon Rnd1 binding (Tong et al., 2009) or in its RapGAP activity (Wang et al., 2012).

Plexin-B proteins can regulate RhoA activity, although they do not interact with RhoA. Plexin-B1 and plexin-B2 can either activate RhoA activity through their interaction with RhoGEFs, or inhibit RhoA activity through Grb2-mediated recruitment of p190RhoGAP (Azzarelli et al., 2014; Barberis et al., 2005; Hota and Buck, 2012; Perrot et al., 2002; Sun et al., 2012). Neither plexin-B1 nor plexin-B2 cytoplasmic domains significantly altered RhoA activity, when normalized to total RhoA, either with or without Rnd1 or Rnd3 co-expression (Fig. 3F). Interestingly, Rnd1 and plexin-B2 independently reduced the level of total RhoA, which reduced the amount of RhoA-GTP in cells (normalized to GAPDH) (Fig. 3G). RhoA is known to be targeted for ubiquitin-mediated degradation (Visvikis et al., 2010) and it is possible that Rnd1 and plexin-B2 influence this process.

### The Rnd3 C-terminus and effector domain are both required for plexin-B2 association

**Fig. 4.**
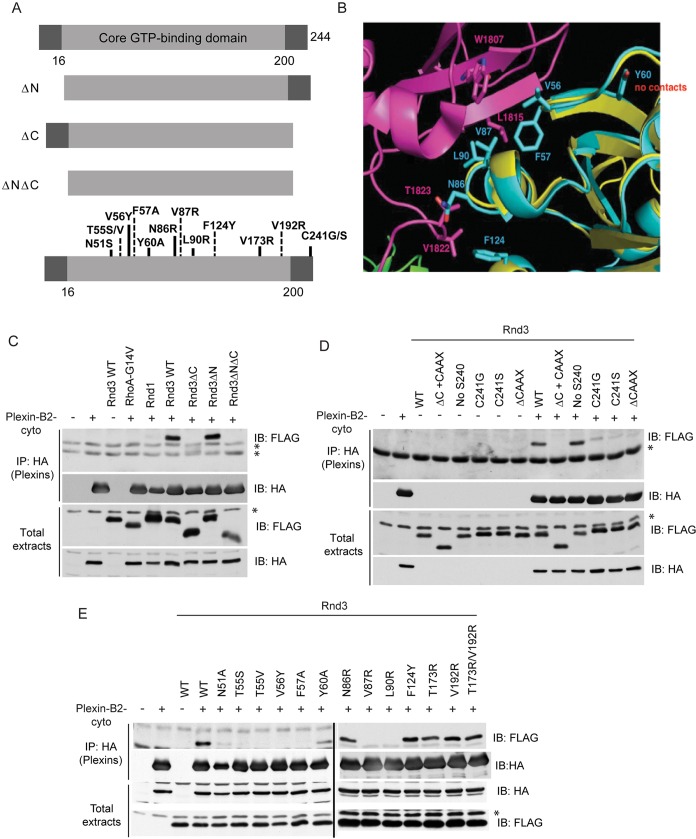
**Rnd3 C-terminus and effector domains are required for plexin-B2 interaction.** (A) Schematic representation of Rnd3 mutants used in this study. Full-length Rnd3 has N- and C-terminal extensions and a core GTP-binding region (residues 16–200). Rnd3ΔC contains amino acids 1–200, Rnd3ΔN amino acids 16–244 and Rnd3ΔNΔC amino acids 16–200. Point mutants are shown below. (B) Structure prediction analysis of the key residues involved in Rnd3 interaction with plexin-B RBD is represented as a ribbon diagram identifying Rnd3 effector domain and non-effector domain amino acids. Plexin-B1 RBD is shown in pink, Rnd1 in yellow (PDB 2REX), Rnd3 in cyan. (C–E) Lysates of COS7 cells expressing HA–plexin-B2-cyto and FLAG–Rnd1, FLAG–Rnd3 mutants or FLAG–RhoA-G14V were immunoprecipitated with anti-HA antibody followed by immunoblotting with the indicated antibodies. All blots are representative of three independent experiments; * indicates IgG or nonspecific bands.

Because Rnd3 interacts preferentially with plexin-B2, we characterized the regions of Rnd3 required for plexin-B2 interaction, using deletion mutants and specifically designed point mutants (Fig. 4A), which were chosen on the basis of modelling predictions. The crystal structure of the Rho-binding domain of plexin-B1 with Rnd1 (PDB: 2REX) indicates that amino acids in the ‘effector loops/switch 1 and switch 2 regions’ of Rnd1 are required for plexin-B1 interaction (Tong et al., 2009; Wang et al., 2011). Modelling of Rnd3 into this crystal structure allowed us to identify potential amino acids required for Rnd3–plexin-B2 binding (Fig. 4B; model kindly provided by David Komander).

Deletion of the Rnd3 N-terminus (amino acids 1-16; Rnd3ΔN) did not affect the plexin-B2–Rnd3 interaction, whereas deletion of the Rnd3 C-terminus (amino acids 200-244; Rnd3ΔC) abrogated plexin-B2–Rnd3 binding (Fig. 4C). Rnd3 has a CAAX box at the C-terminus that is required for farnesylation and membrane localization (Foster et al., 1996). Mutations of the Rnd3 CAAX box, including Rnd3-C241G, Rnd3-C241S and Rnd3-ΔCAAX (last four amino acids deleted), strongly reduced Rnd3 interaction with plexin-B2 (Fig. 4D). However, the Rnd3 CAAX box was not sufficient for plexin-B2 interaction, as shown by the fact that Rnd3-ΔC with the Rnd3 CAAX box (ΔC+CAAX) showed only very weak interaction with plexin-B2. This indicates that amino acids in the C-terminal region upstream of the CAAX box are required for the plexin-B2–Rnd3 interaction, as well as Rnd3 membrane localization.

Rnd3 amino acids T55, V56, F57, Y60, V87 and L90 in the effector loops were required for its interaction with plexin-B2, whereas N51 and N86 were not required (Fig. 4E). Based on our structural model (Fig. 4B), these results imply that Rnd3 interacts with the RBD of plexin-B2 in a similar way to Rnd1 interaction with the plexin-B1 RBD, but that additional C-terminal residues in Rnd3 are likely to be required to confer the specificity of Rnd3 for plexin-B2.

### Plexin-B2-Rnd3 interaction does not require Rnd3 phosphorylation

**Fig. 5.**
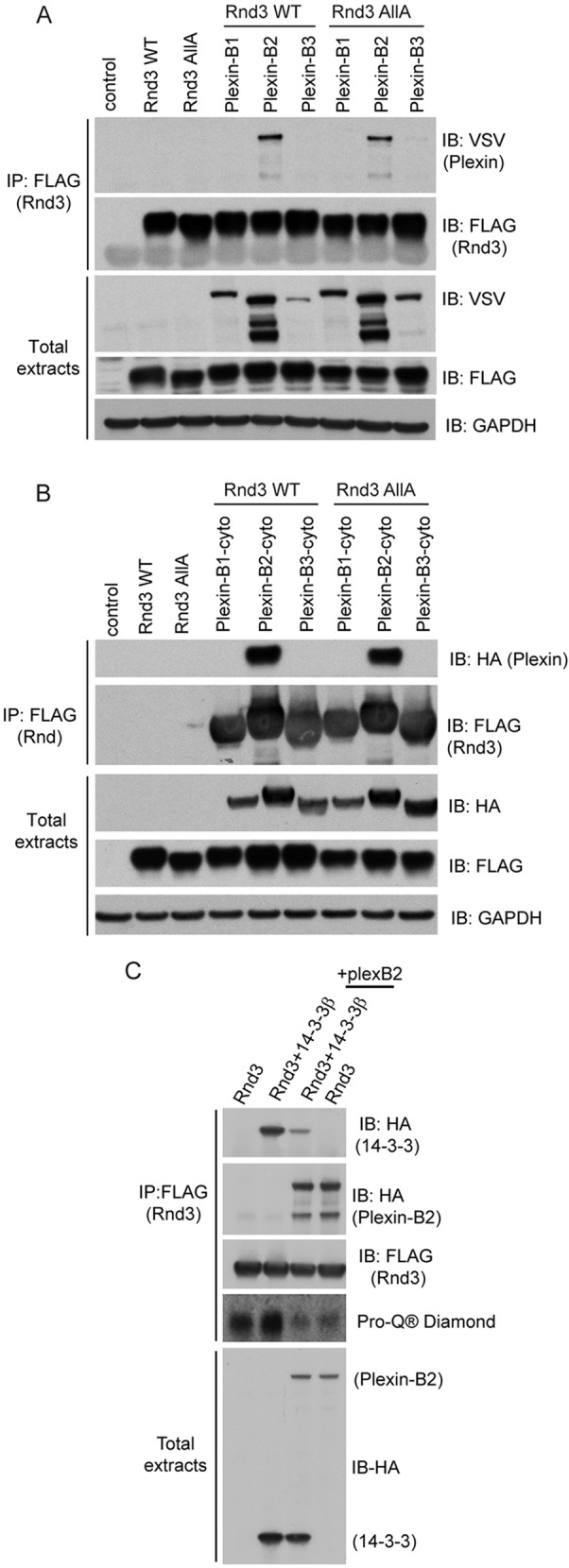
**Rnd3 phosphorylation is not required for plexin-B2 interaction.** (A,B) Lysates from COS7 cells expressing full-length VSV-tagged plexins B1, B2 or B3 (A) HA-tagged plexins B1, B2 or B3-cyto (B) and FLAG–Rnd3 or FLAG–Rnd3-AllA were immunoprecipitated with anti-FLAG antibody, followed by immunoblotting. (C) COS7 cells expressing HA–plexin-B2-cyto, FLAG–Rnd3 and/or HA–14-3-3β were lysed and lysates immunoprecipitated with anti-FLAG antibody, followed by immunoblotting with the indicated antibodies. All blots are representative of three independent experiments.

Rnd3 is phosphorylated by ROCK1 and PKC and has seven known phosphorylation sites, which include S7 and S11 located at the N-terminus and five residues located in the C-terminal region (Fig. 4A) (Madigan et al., 2009; Riento et al., 2005). Rnd3 interaction with ROCK1 leads to Rnd3 phosphorylation, and requires Rnd3 amino acids T173 and V192 (Komander et al., 2008). To establish whether Rnd3 phosphorylation might regulate plexin-B2 binding, plexin-B2 was co-expressed with wild-type Rnd3 or Rnd3-AllA, in which all seven serine/threonine phosphorylation sites are replaced by alanine (Riento et al., 2005). Plexin-B2 (full-length and cytoplasmic region) bound equally well to wild-type Rnd3 or Rnd3-AllA (Fig. 5A,B), indicating that the interaction is not dependent on Rnd3 phosphorylation. As described above, very little plexin-B3 or plexin-B1 associated with either wild-type Rnd3 or Rnd3-AllA (Fig. 5A,B), consistent with the preference of Rnd3 for plexin-B2 binding (Fig. 2).

We have previously reported that Rnd3 binds to 14-3-3 proteins, which is dependent on Rnd3 C-terminal phosphorylation and farnesylation (Riou et al., 2013). We tested whether 14-3-3 proteins and plexin-B2 could simultaneously form a complex with Rnd3. Rnd3 co-immunoprecipitation with 14-3-3β was decreased when plexin-B2-cyto was co-expressed in cells, indicating that plexin-B2 and 14-3-3β interaction with Rnd3 are mutually exclusive (Fig. 5C). As previously observed (Riou et al., 2013), Rnd3 phosphorylation was increased when 14-3-3β was co-expressed, because 14-3-3 binds to phosphorylated Rnd3 and presumably protects it from dephosphorylation. However, in the presence of plexin-B2, Rnd3 phosphorylation was decreased, indicating that plexin-B2 is likely to bind preferentially to unphosphorylated Rnd3. This implies that Rnd3 has to be dephosphorylated to induce its dissociation from 14-3-3 protein, which then allows it to bind to plexin-B2.

### Interaction of Rnd3 with plexin-B2 stimulates Rnd3-induced rounding and enhances Rnd3 inhibition of invasion

Previous studies have shown that stimulation of plexin-B1 by its ligand Sema4D and by Rnd1 can promote cell contraction in COS7 cells and induce growth cone collapse in neuronal cells (Oinuma et al., 2004, 2003). Rnd3 is known to induce loss of stress fibres and reduce actomyosin contractility in a variety of cell types (Riou et al., 2010). We therefore investigated whether plexin-B2 interaction with Rnd3 affected cell morphology.

**Fig. 6.**
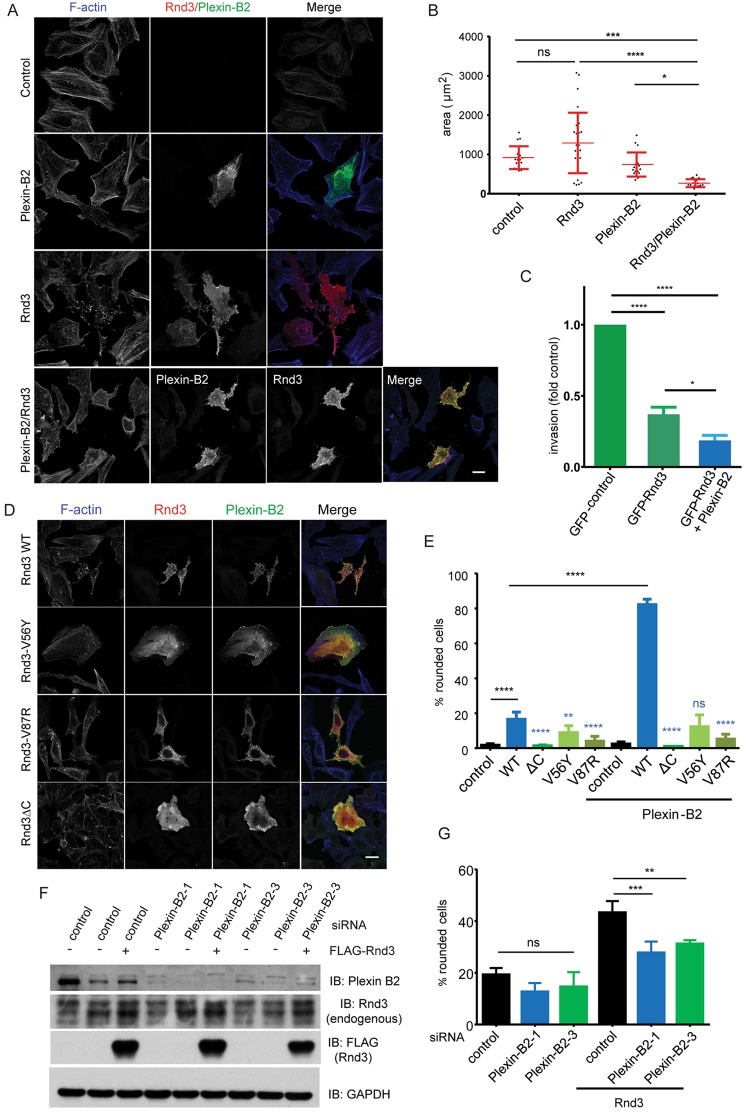
**Plexin-B2 enhances Rnd3-induced cell rounding and inhibition of invasion.** (A,B) HeLa cells were transfected with pCMV5 (empty vector control), FLAG–Rnd3 and/or full-length plexin-B2. After 16–18 h, cells were fixed and stained with anti-FLAG antibody and anti-plexin-B2 antibody. Actin filaments were visualized with AlexaFluor-546- or AlexaFluor-633-conjugated phalloidin. Scale bar: 10 µm. (B) Cell spread area (mean±s.e.m., *n*=3). Each dot represents a single cell; 19 cells were analysed for each condition. (C) HeLa cells were transfected with GFP (control), or GFP–Rnd3 with or without plexin-B2. Cell invasion was measured using Matrigel-coated Transwell filters. Invasion is shown relative to GFP-expressing control cells (mean±s.e.m., *n*=35). (D,E) HeLa cells (1×10^5^ cells/ml) were transfected with pCMV5 (control), full-length plexin-B2 and wild-type (WT) FLAG–Rnd3 or FLAG–Rnd3 mutants that do not bind to plexin-B2 (Rnd3-V56Y, Rnd3-V87R and Rnd3-1–200). After 24 h, cells were fixed and stained for plexin-B2 (green) and FLAG–Rnd3 proteins (red). Actin filaments were detected with AlexaFluor-633-conjugated phalloidin (blue). Scale bar: 10 μm. (E) Mean percentage±s.e.m. of rounded cells from three independent experiments; *n*≥100 cells per experiment. (F) HeLa cells were transfected with two siRNAs targeting plexin-B2 or with control siRNA. After 48 h, cells were transfected with FLAG-tagged Rnd3. Cells were lysed or fixed, permeabilized and immunostained 24 h later. Plexin-B2 depletion was determined by immunoblotting with anti-plexin-B2 antibody. Endogenous Rnd3 and exogenous Rnd3 were detected using anti-Rnd3 or anti-FLAG antibody, respectively. Blots are representative of three independent experiments. (G) Mean percentage±s.e.m. of rounded cells from three independent experiments; *n*≥100 cells per experiment. For all graphs, *P<0.05, **P<0.01, ***P<0.001 ****P<0.0001; ns, not significant; one-way ANOVA with Tukey posthoc test.

HeLa cells were transfected with constructs encoding wild-type Rnd3 and/or full-length plexin-B2. Rnd3 induced loss of actin stress fibres and thin protrusions, as expected (Fig. 6A). Plexin-B2 alone did not induce a phenotype. However, co-expression of plexin-B2 and Rnd3 strongly induced cell rounding, measured by a significant decrease in cell spread area (Fig. 6A,B). To test the physiological consequences of these morphological changes induced by Rnd3 and plexin-B2, we investigated their effects on HeLa cell invasion through Matrigel-coated Transwell filters. Rnd3 alone inhibited invasion of HeLa cells, and co-expression of plexin-B2 further reduced invasion (Fig. 6C).

We next tested the effects of Rnd3 mutations that prevent its interaction with plexin-B2 (see Fig. 3E). Rnd3ΔC, Rnd3-V56Y and Rnd3-V87R, which do not bind to plexin-B2, induced much less cell rounding than wild-type Rnd3 when co-transfected with plexin-B2 (Fig. 6D,E). These mutants also did not reduce cell area when co-expressed with plexin-B2, in contrast to wild-type Rnd3 (Fig. S1). Approximately 18% of cells expressing wild-type Rnd3 alone, without co-expressing plexin-B2, induced cell rounding, whereas the Rnd3 mutants induced significantly less cell rounding (Fig. 6E). This indicates that endogenous plexin-B2 is likely to contribute to the response to Rnd3.

These results indicate that plexin-B2 interaction with Rnd3 promotes cell rounding, which correlates with reduced cell invasion. We therefore depleted plexin-B2 to determine whether it is required for Rnd3 responses. Plexin-B2 knockdown by two different siRNAs strongly reduced Rnd3-induced cell rounding but did not affect control cells or alter endogenous Rnd3 expression (Fig. 6F,G; Fig. S2). Taken together, these results indicate that Rnd3 interaction with plexin-B2 is important for it to induce actin remodelling and cell shape change, and define plexin-B2 as a Rnd3 effector.

## DISCUSSION

Rnd proteins are known to bind to plexin-B receptors, but the physiological consequences of these interactions are still unclear. Here, we show that plexin-B2 increases the ability of Rnd3 to induce cell rounding and reduce HeLa cell invasion, and that plexin-B2 depletion reduces Rnd3-induced rounding, consistent with it being an effector for Rnd3. We also compare for the first time the interaction of each of the three Rnd proteins with the three plexin-B proteins. Of the three plexin-B proteins, we find that Rnd3 preferentially interacts with plexin-B2, although it is also able to associate more weakly with plexin-B3 and plexin-B1 in cells.

Of the three plexin-B proteins, the interaction between plexin-B1 and Rnd1 has been the most characterized (Rohm et al., 2000). It was previously reported that the cytoplasmic domain of plexin-B1 bound to all three Rnd family members (Uesugi et al., 2009) and that Rnd1 interacted more weakly with plexin-B2 than plexin-B1 (Oinuma et al., 2003). In our hands, however, Rnd1interaction with all three plexin-B proteins was considerably weaker than that of Rnd2. Sequence alignment of plexin-B3 with that of plexin-B1 suggests that plexin-B3 should interact with Rnd1 (Wang et al., 2011). Our co-immunoprecipitation data indicate that there is a relatively weak association between plexin-B3 and Rnd1. Interestingly, plexin-B2 Rho-binding domain lacks several amino acids and has several amino acid differences compared with plexin-B1 and plexin-B3 (see Fig. 2A), which could explain its preferential interaction with Rnd3. Plexin-B1 dimerizes when it binds to its dimeric Sema4D ligand, and dimerization is required for Sema4D-induced signalling (Janssen et al., 2010). However, plexin-B2 is predicted not to form stable dimers as a result of the presence of transmembrane prolines (Zhang et al., 2015). Signalling by plexin-B2 must therefore be different to that of plexin-B1.

The GAP domain of plexin-B2 is central to its function *in vivo*, although its targets are not clear (Worzfeld et al., 2014). Plexin-B1 has been reported to interact with and act as a GAP for Rap1 as well as R-Ras and its close relative M-Ras (Oinuma et al., 2004; Saito et al., 2009; Wang et al., 2012). Plexin-B2 does not appear to act as a GAP for R-Ras in mice, although Sema4C acts through plexin-B2 to reduce R-Ras activity in HEK293 cells (Worzfeld et al., 2014). However, plexin-B2 and plexin-B3 have not been tested for interactions with R-Ras or Rap1. We found that all three plexin-B family members associate with R-Ras, Rap1A and Rap1B. However, neither plexin-B1 nor plexin-B2 decreased R-Ras or Rap1 activity, with or without Rnd1 or Rnd3 expression. Regulation of Rap1 or R-Ras activity by plexin-B1 normally requires stimulation with its ligand Sema4D (Oinuma et al., 2004; Wang et al., 2012), although this can be autocrine. For example, COS7 cells and cervical cancer cell lines express plexin-B1 and its ligand Sema4D (Damola et al., 2013; Liu et al., 2014), and thus Sema4D is likely to act in an autocrine manner to stimulate plexin-B1. In HeLa cells, we found that plexin-B2 induced some cell rounding in the absence of added ligand, suggesting that they secrete the plexin-B2 ligand Sema4C.

In addition to the GAP domain, plexin-B receptors share a common C-terminal PDZ-binding motif (Pascoe et al., 2015). In plexin-B1 this interacts with PDZ-RhoGEF and LARG to activate RhoA, leading to growth cone collapse (Swiercz et al., 2002). By contrast, Rnd proteins and plexin-B1 can antagonize RhoA signalling by activating p190RhoGAP (Sun et al., 2012; Swiercz et al., 2008; Wennerberg et al., 2003). Plexin-B2 could therefore act as a scaffold, bringing together Rnd3, p190RhoGAP and RhoA. However, in cortical neurons *in vivo*, plexin-B2 was reported to activate RhoA by competing with p190RhoGAP for Rnd3 binding, thereby blocking Rnd3-mediated inhibition of RhoA (Azzarelli et al., 2014). It is clear that the effect of plexins on RhoA activity is context-dependent, for example via its association with either ErbB2 or Met tyrosine kinase receptors (Swiercz et al., 2008). Although RhoA activity was not affected by plexin-B1 or plexin-B2 expression in our experiments, RhoA expression was reduced by Rnd1 or plexin-B2, which could reflect targeting of the inactive form of RhoA for ubiquitination and degradation by a family of RhoA-binding BTB domain adaptors (BACURD proteins) (Chen et al., 2009). A potential interaction of Rnd3 with BACURD3, identified in our yeast two-hybrid screen, might promote degradation of inactive RhoA. Intriguingly, BACURD1 and BACURD2 have recently been reported to interact with Rnd2 and Rnd3 (Gladwyn-Ng et al., 2016).

Our data indicate that both the Rnd3 effector domain and the Rnd3 C-terminal extension are required for interaction with plexin-B2. Additionally, mutation of the C-terminal CAAX box strongly reduced the interaction between Rnd3 and plexin-B2. Because the CAAX box is required for Rnd3 farnesylation and membrane association (Foster et al., 1996), this indicates that the Rnd3 C-terminal extension is most likely to interact with the membrane-proximal region of plexin-B2. This interaction probably confers specificity to the interaction of Rnd3 with plexin-B2 as the C-terminal extensions of Rnd proteins are very different in sequence (Riou et al., 2013). Based on the structure of Rnd1 with the plexin-B1 RBD domain (Tong et al., 2009), the effector domain of Rnd3 interacts with the plexin-B2 RBD. The interaction between plexin-B2 and Rnd3 results in cytoskeletal remodelling to produce a rounded phenotype with decreased spread area. Furthermore, plexin-B2 knockdown strongly reduces Rnd3-induced cell rounding, indicating that plexin-B2 is essential for Rnd3 responses.

We show that the plexin-B2–Rnd3 interaction does not require Rnd3 phosphorylation on any of the seven known phosphorylation sites. By contrast, Rnd3 phosphorylation is required for binding to 14-3-3 proteins, leading to translocation of Rnd3 from membranes to the cytosol and inhibition of Rnd3 cellular function (Riou et al., 2013). Our data indicate that plexin-B2 competes with 14-3-3 for Rnd3 binding, and that the binding of Rnd3 to plexin-B2 and that of Rnd3 to 14-3-3 are mutually exclusive, consistent with 14-3-3 being a negative regulator of Rnd3 signalling.

The phenotypes of Rnd3-deficient mice and plexin-B2-deficient mice strongly suggest a functional relationship between Rnd3 and plexin-B2 *in vivo*. For example, loss of Rnd3 results in profound neurodevelopmental defects (Mocholí et al., 2011), including a significantly thinner granule cell layer in the olfactory bulb (Ballester-Lurbe et al., 2015). Plexin-B2-deficient mice have substantial similarities, including defective migration of granule cell precursors in the olfactory bulb and cerebellum (Deng et al., 2007). By contrast, mice lacking plexin-B1 and plexin-B3 do not have detectable central nervous system phenotypes (Deng et al., 2007; Worzfeld et al., 2009).

In summary we show here that, of the three plexin-B receptors, plexin-B2 binds preferentially to Rnd3, and that plexin-B2 is important for Rnd3-induced cell rounding. This specificity of Rnd3 for plexin-B2 is likely to be mediated by the requirement of the C-terminal domain of Rnd3 for plexin-B2 interaction. It is plausible that plexin-B2 brings Rnd3 and p190RhoGAP together to enhance Rnd3-induced rounding. The role of plexin-B2 as a target for Rnd3 signalling could explain the apparently opposing effects of plexin-B2 in cancer cell invasion and cancer progression: in some cancer types such as breast cancer, both Rnd3 and plexin-B2 appear to inhibit hallmarks of tumorigenesis (Malik et al., 2015; Zhu et al., 2014), whereas in gliomas they promote tumour progression (Clarke et al., 2015; Le et al., 2015).

## MATERIALS AND METHODS

### Antibodies

The following antibodies were from Santa Cruz Biotechnology: mouse monoclonal anti-myc sc-40 (9E10), rabbit polyclonal anti-myc sc-789 (A-14), rabbit polyclonal anti-R-Ras sc-523, mouse monoclonal anti-RhoA sc-418, goat polyclonal anti-plexin-B2 sc-34504 (I-16), mouse monoclonal anti-GST sc-138 (B-14), rabbit polyclonal anti-GFP antibody sc-8334 and mouse monoclonal anti-14-3-3β antibody sc-1657. Mouse monoclonal anti-FLAG F-1804 (M2, Sigma-Aldrich), rabbit polyclonal anti-FLAG F-7425 (Sigma-Aldrich), rat monoclonal anti-HA antibody (3F10) (Roche Applied Science), rabbit polyclonal Rap1A/Rap1B antibody 4938, and rabbit polyclonal Rap1B antibody 2326 (Cell Signaling Technology) were all used at 1:1000 for western blotting (WB). Rabbit polyclonal anti-VSV-G antibody (V4888, Sigma-Aldrich) was used at 1:8000 for WB, mouse monoclonal Rnd3/RhoE antibody (hybridoma supernatant clone 4) (Riento et al., 2003) at 1:5, mouse anti-GADPH antibody (MAB374, Millipore) at 1:10,000 and sheep anti-human plexin-B2 antibody (AF5329, R&D Systems) at 1:2000. Secondary antibodies used for WB were horseradish peroxidase-linked sheep anti-mouse IgG (NA931V), donkey anti-rabbit IgG (NA934V), goat anti-rat IgG (NA935V) (GE-Healthcare) and donkey anti-sheep IgG (HAF016, R&D Systems). Mouse anti-FLAG A2220 (M2, Sigma-Aldrich) and anti-HA (A-2095, Sigma-Aldrich) antibodies bound to agarose were used for immunoprecipitations.

### Expression vectors, cloning and site-directed mutagenesis

Rnd3 deletion mutants 16–244 and 1–200 were amplified from pCMV5–FLAG–Rnd3 (Riento et al., 2003) by PCR and cloned into *Eco*RI and *Hin*dIII sites of pCMV2–FLAG. For Rnd3 point mutants, pCMV5–FLAG–Rnd3 was mutagenized using the Quikchange system (Stratagene). The nucleotide changes were verified by DNA sequencing (Eurofins-MWG, UK). GFP–Rnd3 was a gift from Dr Erik Sahai (Francis Crick Institute, London, UK).

Expression vectors encoding full-length VSV-tagged plexins B1, B2 and B3 were kindly provided by Professor Luca Tamagnone (University of Torino, Italy), Dr Roberta Azzarelli (Francis Crick Institute, London, UK) and Dr Roland Friedel (Friedman Brain Institute, New York, USA), respectively. Plexin-B cytoplasmic domains were generated by PCR of human plexin-B1 (residues R1512–L2135), plexin-B2 (residues R1225–L1841), and plexin-B3 (residues R1277–L1909) with N-terminal HA tags, and cloned into pcDNA3 *Kpn*I–*Not*I (plexin-B1 and plexin-B2) or *Kpn*I–*Eco*RI sites (plexin-B3). Expression vectors encoding Myc-tagged R-Ras and Ras-binding domain of Raf (amino acids 55–131) were kindly provided by Dr Annette Self and Professor Chris Marshall (Institute of Cancer Research, London, UK). The Rap-binding domain of RalGDS (amino acids 788–884) and HA-tagged Rap1B were gifts from Professor Johannes Bos (University Medical Centre, Utrecht, Netherlands) and GFP-tagged Rap1A was from Professor Nancy Hogg (Francis Crick Institute, London, UK).

### Yeast two-hybrid screening

The yeast two-hybrid screen was carried out essentially as previously described (McKenzie et al., 2006), using mouse Rnd3 lacking the C-terminal CAAX box cloned into pGBT9 to encode the GAL4 DNA-binding domain fused to Rnd3 as bait (Riento et al., 2003). A Human Lung Matchmaker cDNA library in pACT2 (Clontech), which produces proteins fused to the GAL4-activation domain, was used to screen for proteins that interact with Rnd3. A β-galactosidase filter assay was used to identify yeast colonies expressing potential Rnd3 interacting partners.

### Cell culture and transfection

COS7 cells (obtained from Michael Waterfield, Ludwig Institute for Cancer Research) and HeLa cells (obtained from Michael Way, Francis Crick Institute, London, UK) were grown in Dulbecco's modified Eagle's medium (DMEM) containing 10% foetal calf serum (FCS). Cell lines were not authenticated in our laboratory. For transfection, COS7 cells were washed with 5 ml of cold electroporation buffer (120 mM KCl, 10 mM K_2_PO_4_, 10 mM KH_2_PO_4_ pH 7.6, 25 mM HEPES pH 7.6, 2 mM MgCl_2_, 0.5% Ficoll). The buffer was removed and cells were re-suspended in 250 µl of cold electroporation buffer and electroporated at 250 V and 960 µF with 5 µg of DNA. The cells were then plated onto 10-cm diameter dishes and incubated for 24 h prior to lysis. HeLa cells were transiently transfected with plasmids using Lipofectamine 2000 (ThermoFisher Scientific).

siRNAs were from Dharmacon (GE Healthcare): siplexin-B2 siRNA1 (GCAACAAGCUGCUGUACGC), siplexin-B2 siRNA3 (UGAACACCCUCGUGGCACU) and ON-TARGET siControls (D-001810-01, D-001810-02). Growing HeLa cells were transfected with 100 nM plexin-B2 siRNAs or control siRNA using Oligofectamine (ThermoFisher Scientific). After 48 h, HeLa cells were further transfected with pCMV5–FLAG–Rnd3 using Lipofectamine 2000. HeLa cells were fixed 24 h later (72 h after siRNA transfection) and stained for confocal microscopy analysis.

### Immunoprecipitation and immunoblotting

COS7 or HeLa cells were lysed 16–18 h after transfection with ice-cold cell lysis buffer (20 mM Tris-HCl pH 8, 130 mM NaCl, 1% Triton X-100, 1 mM DTT, 10 mM NaF, 1 mM phenylmethylsulfonyl fluoride, 10 μg/ml aprotinin, 10 μg/ml leupeptin, 0.1 mM sodium orthovanadate). After centrifugation, the supernatants were incubated with mouse anti-HA antibody or mouse anti-FLAG antibody bound to agarose beads (20 μl of a 50% slurry) and incubated for 2 h at 4°C. The beads were washed extensively with lysis buffer.

For immunoblotting, proteins were resolved by SDS–PAGE and transferred to nitrocellulose membranes (Schleicher and Schuell). Membranes were blocked with TBS (20 mM Tris-HCl pH 7.6, 137 mM NaCl) containing 5% non-fat dried milk and 0.05% Tween-20, and then incubated with primary antibodies. Bound antibodies were visualized with horseradish peroxidase-conjugated secondary antibodies and enhanced chemiluminescence (ECL; Amersham Pharmacia Biotech).

### GST pull-downs and GTPase activity assays

GST–Ras-binding domain of Raf (de Rooij and Bos, 1997), GST–Rap-binding domain of RalGDS (Franke et al., 1997) and GST–Rho-binding domain of Rhotekin (amino acids 7–89) (Ren et al., 1999) (GST–RBDs) were produced by lysing bacterial pellets in cold STE buffer (10 mM Tris pH 8.0, 150 mM NaCl, 1 mM EDTA, 1 mM phenylmethylsulfonyl fluoride) and homogenizing three to five times using a 19-gauge needle. Lysozyme (100 μg/ml) was added to the homogenate and the mixture incubated for 15 min on ice. DTT (5 mM) was added followed by Tween-20 (1%) and SDS (0.03%). The homogenate was centrifuged at 13,000 ***g*** at 4°C for 30 min. The supernatant was incubated with gluthathione–Sepharose beads for 2 h at 4°C. Beads were then washed in STE buffer followed by Mg^2+^ buffer (25 mM HEPES pH 7.5, 150 mM NaCl, 1% NP-40, 10 mM MgCl_2_, 1 mM EDTA, 25 mM NaF, 1 mM Na_3_VO_4_, 1 mM phenylmethylsulfonyl fluoride, 10% glycerol and Roche protease inhibitor cocktail).

For GST–Rnd3 and GST pull-downs, transfected COS7 cells were lysed in lysis buffer (1% Triton X-100, 20 mM Tris-HCl pH 8, 130 mM NaCl, 10 mM NaF, 1% aprotonin, 10 µg/ml leupeptin, 1 mM dithiothreitol, 0.1 mM Na_3_VO_4_ and 1 mM phenylmethylsulfonylfluoride). Insoluble material was removed by centrifugation and the cell lysates were incubated for 2 h at 4°C with the recombinant GST–fusion proteins on glutathione–Sepharose beads. Bound proteins were analysed by immunoblotting.

For GTPase activity assays, COS7 cells were transfected with plasmids encoding R-Ras, Rap1A, Rap1B or RhoA and incubated for 16–18 h. The cells were lysed in pull-down lysis buffer (25 mM HEPES pH 7.5, 150 mM NaCl, 1% NP-40, 10 mM MgCl_2_, 1 mM EDTA, 25 mM NaF, 1 mM Na_3_VO_4_, 1 mM phenylmethylsulfonyl fluoride, 10% glycerol and Roche protease inhibitor cocktail). Cell lysates were clarified by centrifugation. Supernatants were incubated with GST–RBDs on glutathione–Sepharose beads at 4°C for 2 h. Bound proteins were analysed by SDS–PAGE followed by immunoblotting with rabbit anti-R-Ras antibody, rabbit anti-Rap1A/B or mouse anti-RhoA antibodies.

### Immunofluorescence and confocal microscopy

HeLa cells (1×10^5^ cells/ml) were fixed with 3.7% paraformaldehyde in PBS for 15 min, permeabilized with 0.2% Triton X-100 and incubated for 1 h with anti-plexin-B2 antibody (1:50) to detect plexin-B2 proteins, followed by AlexaFluor-488-conjugated donkey anti-goat antibodies (A11055) or mouse anti-FLAG antibody (1:200) to detect FLAG–Rnd3 proteins, followed by AlexaFluor-546-conjugated donkey anti-mouse antibody (A21202; Molecular Probes/ThermoFisher Scientific). Actin filaments were localized by incubating cells with AlexaFluor-546–phalloidin (A22283; 1:200) or AlexaFluor-633–phalloidin (A22284; 1:200). Coverslips were mounted with mounting medium (Dako) and images were generated with a Zeiss LSM510 confocal microscope using a 63×1.3 NA objective and Zen software. Cell area was measured using ImageJ. Rounded cells were quantified, and graphs generated using Prism (GraphPad software).

### Invasion assay

Hela cells were transfected with plasmids encoding GFP (control), GFP–Rnd3 with or without VSV-tagged full-length plexin-B2 using Lipofectamine 2000 (ThermoFisher Scientific). The upper chambers of Biocoat Matrigel invasion chambers (Corning; 8-µm pore size) were rehydrated with 300 µl of serum-free medium for 2 h at 37°C. HeLa cells (2×10^5^ for each condition) in 0.1% FCS were added to the upper chamber, and medium containing 10% FCS was used as a chemo-attractant in the lower chamber. After 21 h, cells in Transwell inserts were fixed with 3.7% paraformaldehyde for 15 min, and GFP-expressing cells on the top and bottom of the filter were detected using a Zeiss LSM510 confocal microscope and Zen software. Z-stacks (2.03 µm spacing) were acquired for 6–10 fields using a 20× objective (0.5 NA). Reflectance was used to identify the position of the Transwell filter. Invading cells were quantified from three independent experiments. Graphs were generated using Prism (GraphPad Software).

### Statistical analysis

Cell area and cell rounding data, and western blot data, were analysed using one-way ANOVA with Tukey posthoc test for multiple comparisons.
